# New Insights on the Regulation of Glucosinolate Biosynthesis *via* COP1 and DELLA Proteins in *Arabidopsis Thaliana*

**DOI:** 10.3389/fpls.2021.680255

**Published:** 2021-07-01

**Authors:** Henning Frerigmann, Ute Hoecker, Tamara Gigolashvili

**Affiliations:** ^1^Department of Plant-Microbe Interactions and Cluster of Excellence on Plant Sciences, Max Planck Institute for Plant Breeding Research, Cologne, Germany; ^2^BioCenter, Botanical Institute and Cluster of Excellence on Plant Sciences, University of Cologne, Cologne, Germany

**Keywords:** COP1/SPA, DELLA, gibberellin, glucosinolate, jasmonate, MYC2, MYC3, MYC4

## Abstract

The biosynthesis of defensive secondary metabolites, such as glucosinolates (GSLs), is a costly process, which requires nutrients, ATP, and reduction equivalents, and, therefore, needs well-orchestrated machinery while coordinating defense and growth. We discovered that the key repressor of light signaling, the CONSTITUTIVE PHOTOMORPHOGENIC 1/SUPPRESSOR OF PHYTOCHROME A-105 (COP1/SPA) complex, is a crucial component of GSL biosynthesis regulation. Various mutants in this COP1/SPA complex exhibited a strongly reduced level of GSL and a low expression of jasmonate (JA)-dependent genes. Furthermore, *cop1*, which is known to accumulate DELLA proteins in the dark, shows reduced gibberellin (GA) and JA signaling, thereby phenocopying other DELLA-accumulating mutants. This phenotype can be complemented by a dominant gain-of-function allele of *MYC3* and by crossing with a mutant having low DELLA protein levels. Hence, SPA1 interacts with DELLA proteins in a yeast two-hybrid screen, whereas high levels of DELLA inhibit MYC function and suppress JA signaling. DELLA accumulation leads to reduced synthesis of GSL and inhibited growth. Thus, the COP1/SPA-mediated degradation of DELLA not only affects growth but also regulates the biosynthesis of GSLs.

The loss of the COP1/SPA complex affects signaling downstream of DELLAs, attenuates jasmonate response, decreases MYC activity, and reduces glucosinolate levels in Arabidopsis.

## Introduction

Recent years have seen the reintroduction of ecological theories, focusing on the interplay between growth and immunity, into studies on molecular plant biology (Züst et al., 2011; Yang et al., 2012; Campos et al., 2016; Kliebenstein, 2016; Major et al., 2020). While the knowledge on the regulation of defense metabolites is further expanded, the coordination of synthesis of these compounds with other needs of plants still requires more attention. Jasmonate (JA) coordinates immune and growth responses to increase plant survival upon changing environmental cues. Research on the effects of phyB inactivation on JA signaling suggests that the effects of JA on growth and defense can use partially divergent signaling elements and, therefore, are not directly linked in a cause-effect relationship (Howe et al., 2018; Ballaré and Austin, 2019). Light is not only a source of energy but is also an important signal for resource allocation (Huot et al., 2014). As such, the presence of other plants competing for light [sensed by a low red (R) to far-red (FR) light ratio] triggers rapid elongation growth and consumes metabolic resources that could otherwise be invested in the production of defense compounds. Plants have evolved adaptation mechanisms for mediating the balance in the “dilemma to grow or defend.” The phytohormones JA and gibberellin (GA) are fundamentally important in facilitating the rapid adjustment of plant responses to the changing environment (Hou et al., 2013).

Recent studies suggest that the hormone-linked transcriptional network is hardwired to attenuate growth upon activation of JA signaling, and thus, the growth–defense antagonism is not caused by constraints on the availability of metabolic resources that fuel growth and defensive processes (Campos et al., 2016). This was revealed by a suppressor screen of the *jazQ* (*jazQ* being defective in JAZ1, JAZ3, JAZ4, JAZ9, and JAZ10) Arabidopsis mutant, in which the growth–defense trade-off was uncoupled. The *jazQ* mutant exhibits constitutive growth–defense antagonism (reduced growth with enhanced defense). The *jazQ phyB* mutant is morphologically larger than *jazQ* and Col-0 but retains the high level of insect resistance of *jazQ* (Campos et al., 2016).

While Campos et al. (2016) provide evidence that PIF and MYC transcription factors (TFs) are simultaneously activated in *phyB jazQ*, the role of DELLA in this process is still highly debated and is worth having a closer look at. Arabidopsis genome encodes five DELLA proteins with distinct but redundant functions: GAI, RGA, RGL1, RGL2, and RGL3. Contrary to the ability of the *phyB* mutations to completely uncouple the mild growth-defense phenotypes in *jazQ, phyB* null alleles only weakly alleviated the growth and reproductive defects in the *jazD (jaz1–jaz7, jaz9, jaz10, and jaz13)* mutants (Major et al., 2020). The molecular mechanism of JA-induced biomass reduction therefore continues to be intensely studied. Some recent literature studies (Major et al., 2020; Ortigosa et al., 2020) suggested that MYC2 targeting other genes can be involved in this process.

**Figure 1:**
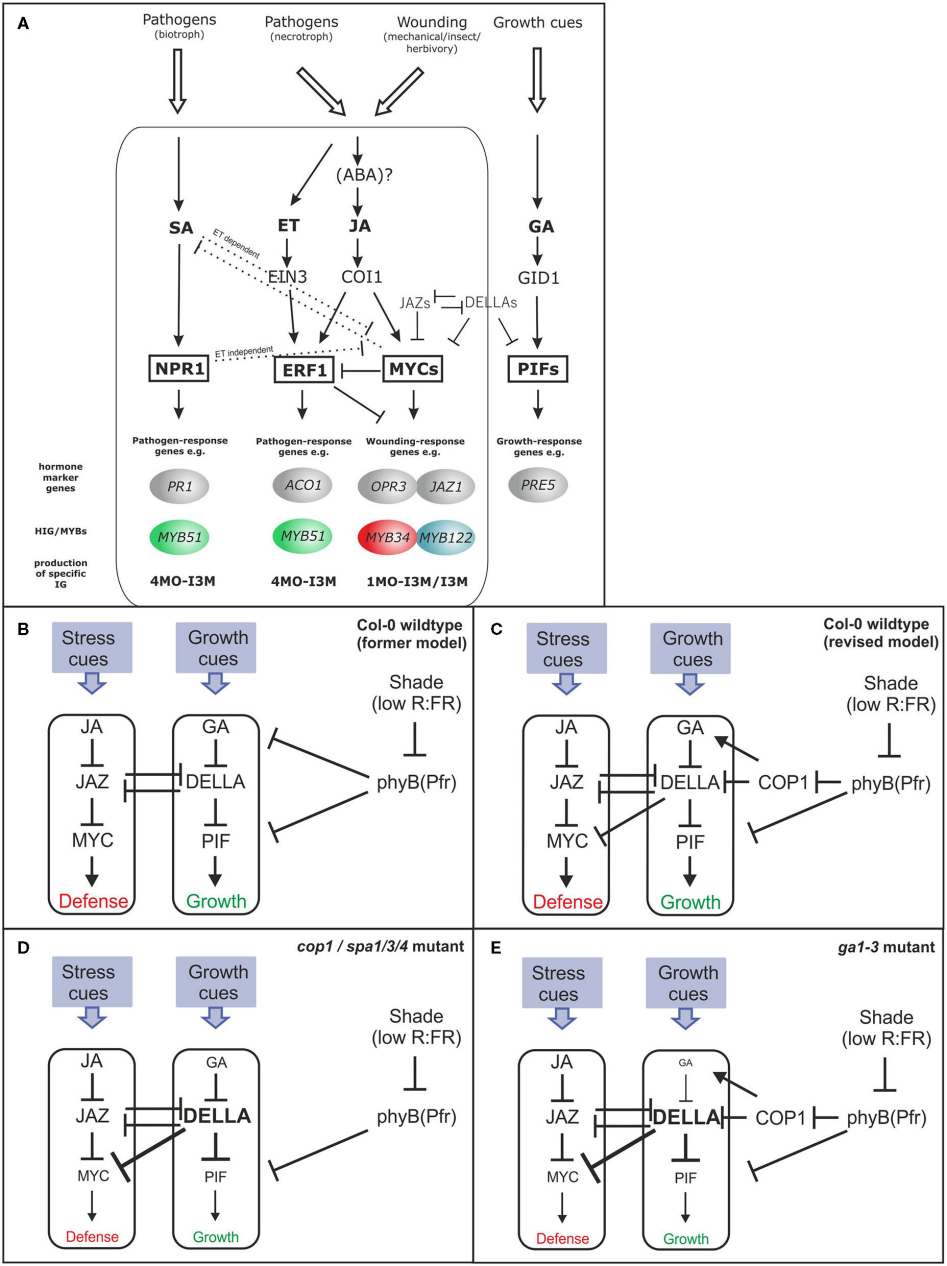
Model for the hormonal signaling network in glucosinolate (GSL) regulation **(A)** and simplified model linking growth and defense trade-off **(B–E)**. The figure depicts the postulated simplified hormonal signaling cascades in *Arabidopsis thaliana* in response to different pathogens involved in the regulation of the HIG-MYBs and the corresponding hormone marker genes. Different biotic/abiotic challenges activate different phytohormone signaling cascades and, thereby, a specific combination of HIG/MYB transcription factors (TFs), which, in turn, led to the production of a specific mixture of IGs [altered from Frerigmann (2016)]. **(B–E)** Describes a simple model of the JA–GA signaling network that governs growth and defense (Campos et al., 2016) **(B)**. In the updated model, COP1 is positioned between phyB and GA signaling. COP1 interacts with DELLA proteins and represses their activity in the dark and additionally affects the GA level probably *via* HY5 repression (Weller et al., 2009; Blanco-Touriñán et al., 2020) **(C)**. These DELLA proteins positively affect JA signaling by interacting with JAZs, but the repression of MYCs is more relevant following strong DELLA accumulation in *cop1, spa1/3/4* **(D)**, or *ga1–3* **(E)** as postulated in this study. **(C)** Provides a revised model in which overaccumulation of DELLAs in *cop/spa* mutants is responsible for the low GLS phenotype. This model contrasts the prevailing model (Navarro et al., 2008; Hou et al., 2010; Fernández-Calvo et al., 2011; Yang et al., 2012), in which JAZ-DELLA interactions cause reciprocal antagonism of GA and JA signaling. This updated model makes clear that the effects of JAZ-DELLA interaction can be overridden, when COP1/SPA regulates the DELLAs, affecting both the growth and biosynthesis of GSLs.

An existing model highlighting the interplay between JA, phyB, and GA (presented in Figure 1B) shows that phyB can perceive changes in the ratio of R:FR light and integrate the shade-avoidance response into GA–JA crosstalk. GA stimulates cell extension growth by promoting the degradation of DELLA proteins that repress PHYTOCHROME-INTERACTING FACTOR (PIF) TFs (Feng et al., 2008). Analogously, JA triggers defense responses *via* the COI1-mediated depletion of JAZ repressors, which interfere with the function of MYC TFs (Fernández-Calvo et al., 2011). In the absence of JA, JAZ repressors bind to MYCs and inhibit the interaction between the MYCs and their targets, attenuating the potential of MYCs to activate the glucosinolate (GSL) pathway genes. JAZ proteins interact directly with DELLA repressors of GA signaling (Hou et al., 2010; Yang et al., 2012); thereby, JA-induced JAZ degradation can modulate the growth-defense balance by increasing the repressive activity of DELLA proteins on growth-promoting TFs. The interaction between JAZ and DELLA proteins prevent these repressors from inhibiting their cognate TFs and enable a reciprocal antagonism between the JA and GA pathways (Hou et al., 2010; Wild et al., 2012).

However, upon light perception, phyB and other photoreceptors inactivate another class of repressors in addition to PIF proteins, which suppress light signaling in dark-grown and shaded Arabidopsis: the CONSTITUTIVE PHOTOMORPHOGENIC 1/SUPPRESSOR OF PHYTOCHROME A-105 (COP1/SPA) complex(es) (Hoecker, 2017). In Arabidopsis, *COP1* is a single-copy gene, whereas SPA proteins are encoded by a small gene family of four genes (*SPA1*–*SPA4*) with overlapping, but partially distinct, functions. The *cop1* and multiple *spa* mutants exhibit constitutive light signaling and show features of light-grown seedlings in complete darkness. The COP1/SPA complex is an E3 ubiquitin ligase that targets positive regulators of light signaling, mainly TFs, for degradation in darkness. Since phyB is an upstream regulator of COP1/SPA and appears to be involved in balancing the JA–GA phytohormone network, we questioned whether the COP1/SPA complex might also be a contributory component. For example, the shade-induced increase in GA level is thought to be regulated *via* shade-induced phyB inactivation [Figure 1B; reviewed by Casal (2013)]. However, the pea ortholog of COP1 is also required to sustain high levels of GA in dark-grown seedlings, probably *via* the ubiquitination of an ELONGATED HYPOCOTYL 5 (HY5) ortholog (Weller et al., 2009). Interestingly, recent findings (Ortigosa et al., 2020) have additionally demonstrated that MYC2, regulating GSLs response, also directly targets the central regulator of photomorphogenesis HY5. In this study, we, therefore, investigated the potential involvement of the COP1/SPA complex in the intricate hormone network that regulates GSL biosynthesis.

Glucosinolates are among the best-studied defense compounds in Arabidopsis, which are also attributed to phytoanticipins and phytoalexins (Piasecka et al., 2015). Despite their constitutive synthesis, plants integrate abiotic and biotic environmental factors with internal signals to increase specific GSL-mediated defense (Burow, 2016). In particular, the relative spatial abundance of indolic GSLs (IGs) and their MYB regulators (*MYB34, MYB51*, and *MYB122*) reflects changes in their response to different stimuli, such as phytohormones (Frerigmann and Gigolashvili, 2014a) or sulfur limitation (Frerigmann and Gigolashvili, 2014b). Most GSL biosynthesis genes have been identified; thus, the regulation of these compounds serves as a model system for secondary metabolites (Burow, 2016; Frerigmann, 2016). The entire GSL biosynthesis pathway is transcriptionally regulated by a group of six homologous MYB TFs and MYC proteins (Frerigmann, 2016; Supplementary Figure 1). These MYC TFs, of which MYC2 is the most prominent member, are also key regulators of JA signaling. Thus, the *myc2/3/4* mutant lacks both the transcription of JA-induced genes, such as *JAZ10* and *VSP2* (Fernández-Calvo et al., 2011) (Figure 1A), as well as the expression of genes involved in the synthesis of IG (such as *CYP79B3* and *CYP83B1*) and aliphatic GSLs (AG) (such as *CYP83A1*) (Frerigmann et al., 2014). However, MYCs regulate GSL biosynthesis not only by interacting with the GSL-MYBs but also due to their central role in JA signaling and, thus, have the capability to activate the expression of IG-MYBs (Figure 1A; Frerigmann, 2016). Along with regulating GSLs, MYCs can also influence leaf development by interfering with the activity of, e.g., PIF4 and by promoting the activity of HY5 (Zhang et al., 2018; Ortigosa et al., 2020).

Thus, *jazQ* shows a specifically elevated level of JA-inducible GSLs such as glucobrassicin (I3M) (Campos et al., 2016), whereas the I3M level in *phyB* is lower (Cerrudo et al., 2017).

In this study, we identified that the COP1/SPA complex dramatically affects the accumulation of GSLs. This result suggests that the absence of the COP1/SPA complex can potentially increase DELLA protein abundance, which in turn, represses GA and JA signaling and inhibits MYC proteins. Low GSL phenotype of *cop1* can be complemented by a dominant gain-of-function allele of *MYC3* and by loss-of-function of DELLA proteins. Thus, COP1/SPA represents a crucial component that integrates light signaling in the hardwired hormone-linked transcriptional network and therefore regulates GSL biosynthesis under changing environmental conditions.

## Materials and Methods

### Plant Growth Conditions and Hormone Treatment

Seeds of *Arabidopsis thaliana* mutant and respective wild-type (Table 1) were stratified for 2–7 days in the dark at 4°C to break seed dormancy. Plants were grown in growth cabinets in a light/dark cycle of 8 h/16 h (9:30–17:30) and a day/night temperature of 21/18°C, 40% humidity, and a mean photon flux density of 120 μmol m^−2^ s^−1^. A minimum of 50 mg of rosette material was harvested from 6-week-old plants in the middle of the day (13:30), was immediately frozen in liquid nitrogen, and kept at −80°C until DNA and RNA extraction or GSL analysis.

**Table 1:** Arabidopsis loss-of-function mutants used in this study.

**Mutant**	**Locus/ATG number**	**Description**	**Publication**	**Comment**
*cop1-4*	COP1, AT2G32950	EMS	Deng and Quail, 1992	Weak allele
*cop1-6*	COP1, AT2G32950	EMS	McNellis et al., 1994	Weak allele
*atr2D*	bHLH05/MYC3, AT5G46760	EMS	Smolen et al., 2002	Constitutively active MYC protein
*cop1-6 atr2D*		EMS	This work	
*ga1-3*	GA1, AT4G02780	EMS	Koornneef and Van der Veen, 1980	Reduced GA biosynthesis
*spa1/3/4*		*spa1-7 spa3-1 spa4-1*	Fittinghoff et al., 2006	
	SPA1, AT2G46340	*spa1-7* (SALK_023840)	Fittinghoff et al., 2006	Full knockout
	SPA3, AT3G15354	*spa3-1* (SAIL_569_F08)	Laubinger and Hoecker, 2003	Full knockout
	SPA4, AT1G53090	*spa4-1* (SAIL_590_C11)	Laubinger and Hoecker, 2003	Full knockout
*gai-mimic*		*pGREEN0179*	Willige et al., 2007	Stable GAI protein
*cop1-4/gai/rga*		*cop1-4 gai-td1 rga-29*	Blanco-Touriñán et al., 2020	
	GAI/RGA2, AT1G14920	*gai-td1* (SAIL_82_F06)		
	RGA1, AT2G01570	*rga-29* (SALK_089146)		
*ga1-3/rga/gai*		*gai-t6*	Achard et al., 2006	
		*rga-t2*		
*ga1-3 /rga/gai/rgl1/rgl2*		*rgl1-1*	Achard et al., 2006	
		*rgl2-1*		

For hormone treatment, wild-type and mutant plants were sprayed after 5.5 weeks with a MOCK, 50 μM GA_3_, 50 μM MeJA, or 50 μM MeJA/50 μM GA_3_ treatment. All treatments contained 0.02 % Silwet to enhance the dispersion of the droplets onto the leaves. Plants were sprayed at three time points (10:30, 11:30, and 12:30) with the corresponding treatments and were kept separately under a hood to prevent cross-contamination. Rosette leaves for expression analysis were harvested 1 h after the last spray treatment (13:30). The remaining untreated pots were repeatedly sprayed once daily (10:30) with the hormone treatments and were harvested 3 days later at 13:30 (75 h after first spray treatment) for GSL determination.

### RNA Extraction and Quantitative Real-Time PCR Analysis

Total RNA extraction and qRT-PCR analysis were performed as described by Frerigmann et al. (2012). The relative quantification of expression levels was performed using the comparative delta Ct method, and the calculated relative expression values were normalized to *PP2A* and compared with the expression level in untreated wild-type plants (Col-0 = 1). If not specified in the figure legend, three independent experiments with three biological replicates from independently grown plants were analyzed (refer to Supplementary Table 1 for primer sequences).

### HPLC Analysis of Desulpho-GS

The isolation and analysis of GSL content were performed using the desulpho-GSL method (Thies, 1979) on an ultra-performance liquid chromatography (UPLC) device (Waters, Eschborn) as described by Gigolashvili et al. (2012).

### Yeast Two-Hybrid Screen

To identify SPA1 interactors, the REGIA yeast two-hybrid TF library (Paz-Ares et al., 2002) was screened using SPA1–pDEST32 as bait. The screen was performed as described by Maier et al. (2013).

## Results

### The COP1 and SPA Protein Complex Has a Pivotal Role in GSL Biosynthesis

**Figure 2:**
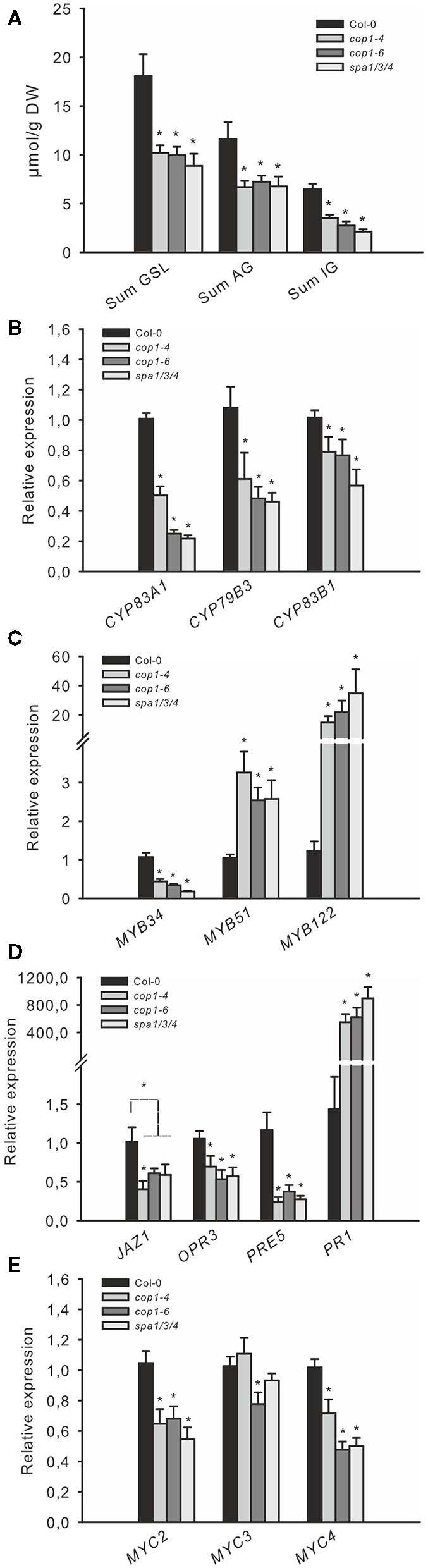
The COP1/SPA complex regulates jasmonate (JA) signaling-dependent GSL regulation. The levels of *GSL* **(A)** and the expression of related genes **(B–E)**, in leaves of short day (SD)-grown 6-week-old *cop1*and *spa1/3/4* mutants. **(A)** The total contents of IGs and AGs, the four main aliphatic GSLs (3MSOP, 4MSOB, 5MSOP, and 8MSOO), and the three major indole GSLs (I3M, 4MO-I3M, and 1MO-I3M) are shown as “Sum GSLs,” “Sum AG,” and “Sum IG,” respectively. Data are means ± SE from four independent experiments with four biological replicates each (*n* = 16). Values marked with asterisks differ significantly from Col-0 (Student's *t*-test; *p* < 0.05).

Previous studies have implicated MYB TFs of subgroup 12 (MYB34, MYB51, MYB122 and MYB28, MYB29, and MYB76) as central regulators of GSL biosynthesis (Gigolashvili et al., 2009; Sønderby et al., 2010; Frerigmann and Gigolashvili, 2014a). Furthermore, the complex signaling network upstream of MYBs includes TFs of higher hierarchical order, as well as a battery of different signaling cues and hormones, such as JA, SA, and ET (Figure 1A). It has also been shown that phyB has a central position in defining the balance of the growth–defense trade-off (Campos et al., 2016; Figure 1B). Because many phyB-mediated effects are transmitted *via* the action of the main repressor of light signaling, the COP1/SPA complex, this prompted an investigation on the role of this complex in the production of defense compounds. The accumulation of GSL defense compounds was analyzed in three mutants with defects in *COP1* or *SPA* genes (*cop1*−*4, cop1*−*6*, and *spa1/3/4*). All three mutants showed a similar strong reduction of nearly 50% in AG and IG levels compared with the wild type (Figure 2A), highlighting a dramatic contribution of the COP/SPA complex to GSL production. The strong reduction in the expression of key marker genes (Figure 2B) for AG (*CYP83A1*) and IG biosynthesis (*CYP79B3* and *CYP83B1*) indicated that the reduced biosynthesis of AG and IG could be associated with the low GSL phenotype in these mutants. The transcriptional regulators MYB34, MYB51, and MYB122 [High Indolic GSL(HIG)-MYB TFs] differentially integrate various signals to transcriptionally regulate GSL biosynthesis genes (Frerigmann and Gigolashvili, 2014a,b). Thus, these genes might have an altered expression pattern in *cop1* and *spa* mutants and lead to reduced GSL biosynthesis. The main regulator *MYB34* was transcriptionally downregulated at this developmental stage in *cop1–4, cop1–6*, and *spa1/3/4* compared with wild-type, whereas expression of *MYB51* and MYB122 was upregulated (Figure 2C). The differential role of MYB34 vs. MYB51 and MYB122 in the regulation of IGs is a known phenomenon, which was reported before (Frerigmann and Gigolashvili, 2014a). *MYB51* and *MYB122* were observed to be induced when *MYB34* levels are decreased, which seems to be a way to regulate the production and turnover of specific modified IG (Frerigmann et al., 2016).

### The *cop1* and *spa* Mutants Possess a Reduced Expression of GSL Biosynthesis and JA Signaling Genes

The reduced expression of JA-inducible *MYB34* and elevated expression of ethylene- (ET) and SA-inducible *MYB51* (Frerigmann and Gigolashvili, 2014a) in *cop1* and *spa* mutants indicates a shift in the phytohormone response toward low JA and increased SA and/or ET signaling (Figure 1A). These HIG-MYB TFs interact with a group of MYC TFs to regulate GSL biosynthesis (Schweizer et al., 2013; Frerigmann et al., 2014). However, these MYC proteins (especially MYC2) are also crucial regulators of JA signaling (Fernández-Calvo et al., 2011). The expression of *MYC2* and *MYC4* was about 40% lower in *cop1–4, cop1–6*, and *spa1/3/4* than in wild-type, suggesting that JA signaling was attenuated (Figure 2E). This was further confirmed by the reduced expression of the JA marker genes, *JAZ1* and *OPR3*. Consistent with the known antagonistic crosstalk between JA and SA (Spoel et al., 2003), expression of the SA-marker gene *PR1* was also highly induced (500–800-fold) in all three mutants, suggesting increased SA signaling (Figure 2D), as was expected due to high *MYB51* expression.

Notably, the COP1/SPA complex was recently shown to interact with DELLA proteins *in vivo*, to ubiquitinate them and thereby negatively regulate the abundance of DELLA proteins in Arabidopsis in a light- and temperature-dependent manner (Blanco-Touriñán et al., 2020). Thus, the COP1/SPA-mediated degradation of DELLA is in line with observations made in this study, as the biosynthesis of GSLs is affected in *cop* and *spa* mutants (Figure 2).

In agreement with all these observations, the *cop1–4, cop1–6*, and *spa1/3/4* mutants showed strongly reduced (~80%) expression of the GA marker gene *PRE5*, substantiating the known role of DELLA proteins serving downstream of COP1 as key repressors of GA signaling (Figure 2D). The COP1/SPA complex is stabilized during darkness and ubiquitinates its targets in the nucleus (Hoecker, 2017). The abundance of DELLA proteins oscillates diurnally, with high levels in the afternoon/evening and low levels in darkness; however, imaging of GFP-tagged RGA expression in *cop1–4* mutant seedlings indicated an altered oscillation pattern, with an increased abundance during darkness (Blanco-Touriñán et al., 2020).

### GSL Accumulation Is Inhibited in *ga1–3* and *gai mimic* Mutants

**Figure 3:**
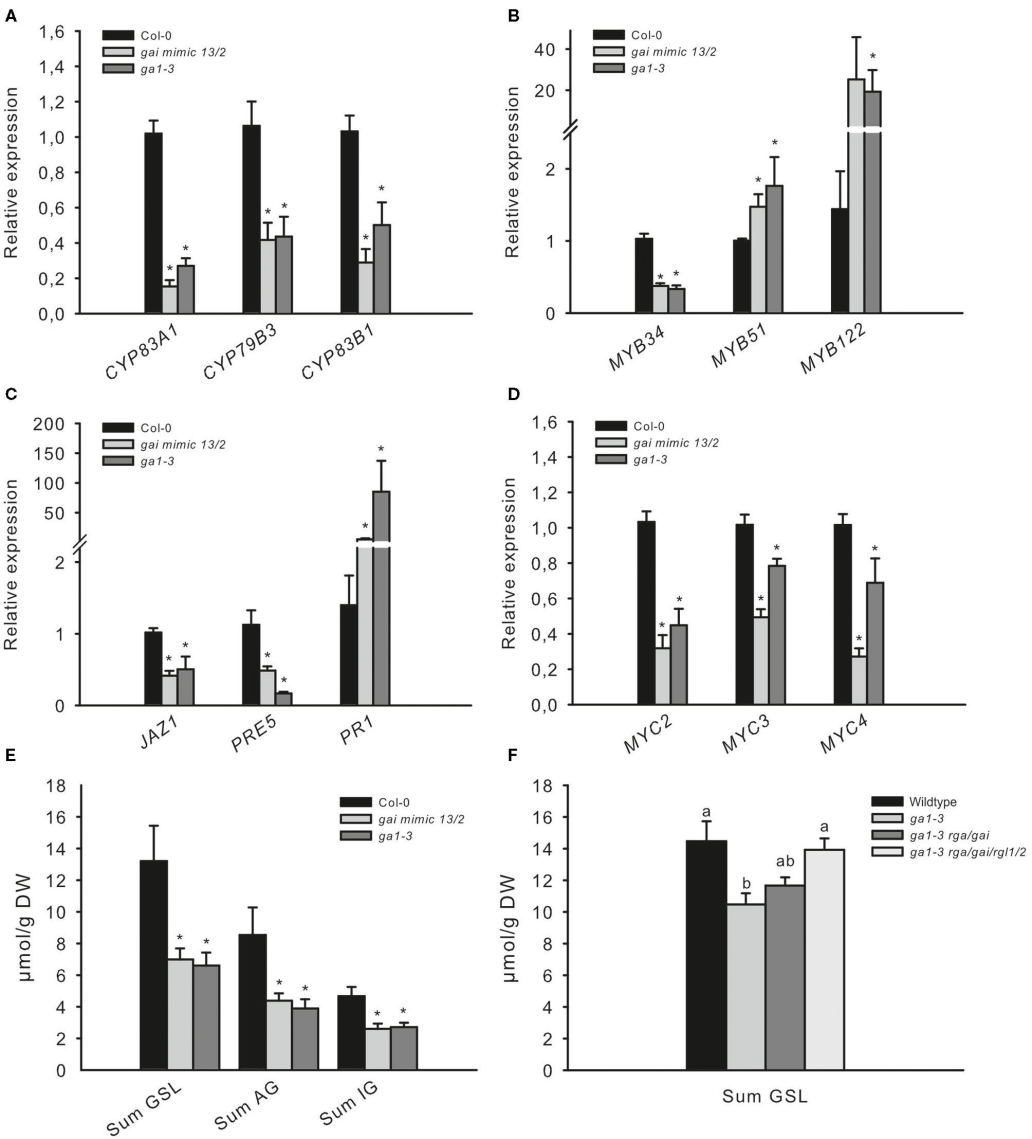
The low GSL chemotype and low JA signaling phenotype in *ga1–3* and *gai mimic* mutants resemble those of the *cop1* mutant. To assess whether high DELLA levels influence the GSL level and related defense gene expression, two DELLA-accumulating mutants were tested. The expression of *GSL* genes **(A)**, *HIG/MYB* regulators **(B)**, hormone marker genes **(C)**, and *MYC* genes **(D)** was investigated in leaves of 6-week-old plants. Data are means ± SE from three independent experiments with three biological replicates in each (*n* = 9). Data for GSLs **(E)** are means ± SE from five independent experiments with four biological replicates in each (*n* = 20). Values marked with asterisks differ significantly from Col-0 (Student's *t*-test; *p* < 0.05). The effect of attenuated DELLA levels in *ga1–3* was tested in 6-week-old plants. Data **(D,F)** are means ± SE from three independent experiments with four biological replicates in each case (*n* = 12). Values marked with different letters differ significantly from each other (the Kruskal–Wallis test followed by a Mann–Whitney pairwise test with Bonferroni-corrected *p*-values, *p* < 0.05).

The COP1/SPA complex affects many aspects of plant growth and development (Hoecker, 2017). To investigate whether the absence of COP1-dependent DELLA ubiquitination and the subsequently increased levels of DELLA proteins during the night are responsible for the observed low JA-signaling and the decreased production of GSL in *cop1–4, cop1–6*, and *spa1/3/4*, we analyzed two other mutants. The *ga1–3* mutant is impaired in GA biosynthesis and thus accumulates DELLA proteins (Silverstone et al., 2001), whereas the *gai mimic* line overexpresses a dominant version of the DELLA protein GAI, which cannot interact with the GID1 receptor and thus accumulates in the cell (Willige et al., 2007). Both mutants, which are expected to accumulate DELLA, exhibited reduced AG and IG levels compared with the wild type and thus resembled the low GSL chemotype (Figure 3E) observed in *cop1–4, cop1–6*, and *spa1/3/4* (Figure 2A). Similarly, both mutants showed the same expression level of GSL biosynthesis genes (Figure 3C), *MYB34, MYB51*, and *MYB122* (Figure 3B); hormone marker genes (Figure 3C); and *MYC* genes (Figure 3D) as the *cop1* and *spa1/3/4* mutants (Figure 2). To further support the hypothesis that the DELLA proteins in *ga1–3* are responsible for the low GSL phenotype, we tested the *ga1–3*-complemented lines *ga1–3/rga/gai* and g*a1–3/rga/gai/rgl1/2*, which contain reduced DELLA levels due to the knockout of respective DELLA-encoding genes (Figure 3F). These mutants exhibited partial complementation of the low GSL phenotype of *ga1–3* plants.

**Figure 4:**
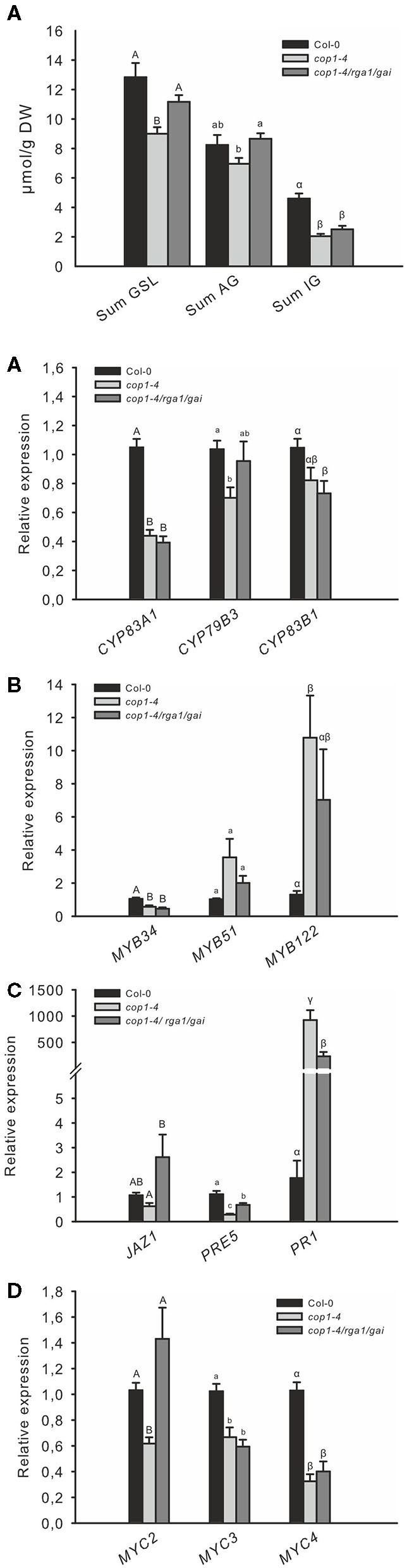
The low GSL level of *cop1* mutant is partially complemented by the absence of DELLAs. The absence of the two DELLA proteins, RGA1 and GAI, in *cop1–4* affects the expression of GSL **(A)** *GSL* genes **(B)**, *HIG/MYB* regulators **(C)**, hormone marker genes **(D)**, and *MYC* genes **(E)**. Data for GSL levels **(A)** are means ± SE from six independent experiments with four to six biological replicates in each case (*n* = 32). Expression data **(B–E)** are means ± SE from three independent experiments with four biological replicates in each case (*n* = 12). Values marked with different letters differ significantly from each other (Kruskal-Wallis test followed by a Mann–Whitney pairwise test with Bonferroni-corrected *p*-values, *p* < 0.05).

These results suggest that the absence of the COP1/SPA complex affects DELLA signaling in cells and represses the response of GA and JA. To test this hypothesis further, we reduced DELLA abundance by knocking out two out of the five DELLA proteins in the *cop1–4* mutant. The absence of only two DELLA proteins was sufficient to partially complement the *cop1–4* phenotype and led to larger leaves, more rosette leaves, and later flowering (Supplementary Figures 2, 3). Correspondingly, the low GSL chemotype in leaves of *cop1–4* was partially complemented in *cop1–4/rga1/gai* (Figure 4A), mainly evident by an elevated AG level. Furthermore, expression of *CYP79B3* was improved in this mutant (Figure 4B), without however significantly affecting the accumulation of IG levels. The absence of *RGA1* and *GAI* in *cop1–4* did not affect the expression of transcriptional regulators *MYB34, MYB51, MYC3*, and *MYC4* and only slightly reduced *MYB122* expression. Notably, the absence of only two out of the five DELLAs partially complemented the reduced expression of GA, JA, and SA marker genes as well as *MYC2* in *cop1–4* (Figures 4D,E). Significantly increased *MYC2* mRNA levels in *cop1–4/gai/rga* vs. *cop1–4* pointed to the substantial role of COP1/SPA in integrating hardwired JA/GA hormone-linked transcriptional network during GSL biosynthesis. Furthermore, this observation suggests that modulations in the DELLA pathway by COP1 knockout (*cop1–4)* resulted in low GA and JA response and high SA signaling.

### DELLAs Play a Central Role Within the JA/GA Hormonal Pathway During GSL Biosynthesis

**Figure 5:**
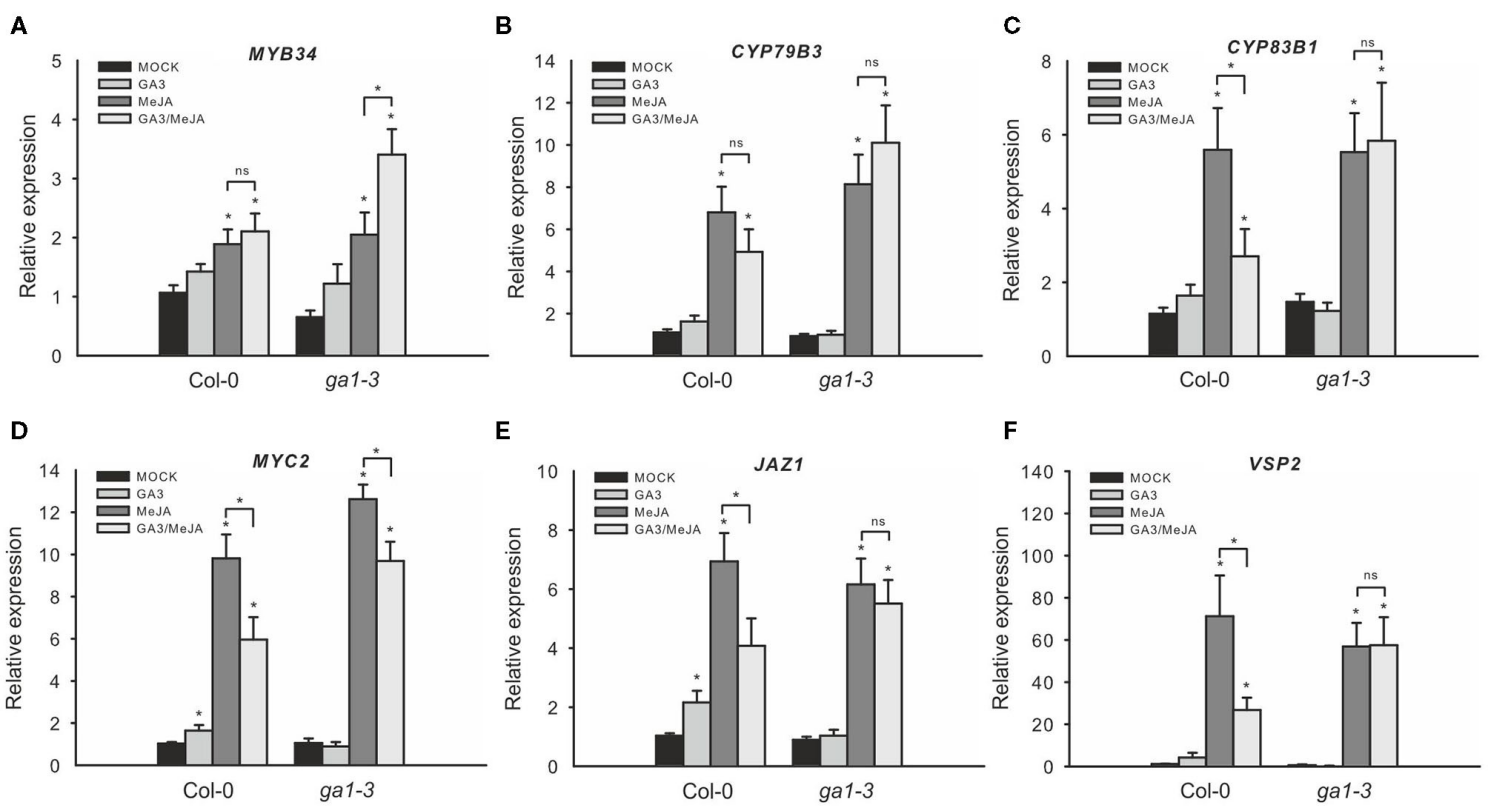
Combined GA/JA treatment does not decrease IG biosynthesis gene expression in the *ga1–3* mutant. The effect of combined GA/JA treatment on gene expression in the DELLA-accumulating mutant *ga1–3* was tested in 6-week-old plants sprayed with MOCK, 50 μM GA_3_, 50 μM MeJA, or 50 μM MeJA/50 μM GA_3_ treatments. *MYB34* **(A)**, *CYP79B3* **(B)**, and *CYP83B1* **(C)** served as marker genes for IG biosynthesis, whereas *MYC2* **(D)**, *JAZ1* **(E)**, and *VSP2* **(F)** served as marker genes for JA signaling. Data are means ± SE from three independent experiments with four biological replicates in each case (*n* = 12). Values marked with asterisks differ significantly from the respective MOCK treatment (Student's *t*-test; *p* < 0.05). Values in parentheses indicate direct comparisons (ns, not significantly different; asterisks mark significantly different values; Student's *t*-test; *p* < 0.05).

The JA and GA signaling pathways are important for the fine-tuning of the growth–defense trade-off: JA triggers the degradation of JAZ proteins and, in turn, de-represses plant defense, whereas GA treatment leads to the degradation of DELLA repressors and increased growth response. The DELLA and JAZ proteins physically interact and mutually repress functions of each other (Hou et al., 2010; Yang et al., 2012). Thus, following a single stress cue, two mechanisms act in concert to ensure that resources are appropriately allocated to either growth or defense. However, it is unclear which signal predominates if both stresses occur simultaneously. To analyze the effect of combined JA/GA treatment on IG biosynthesis, full-grown Col-0 plants were sprayed with these hormones individually or synchronously. Treatment with GA did not markedly affect transcript levels of IG biosynthesis genes or their transcriptional regulators, but *JAZ1* and *MYC2* (two out of the three tested JA marker genes) were slightly upregulated by GA (Figure 5). Treatment with JA strongly induced all tested IG biosynthesis genes, GSL regulators, and JA marker genes. Strikingly, combined GA and JA treatment downregulated most tested JA-inducible genes compared with JA treatment alone. This indicates that the induced degradation of DELLA proteins by GA might counteract activated JA signaling potentially due to the release of previously bound inactivated JAZ proteins, which, then, repress MYC proteins. It was similarly reported that the absence of four DELLAs in the *dellaQ* mutant leads to partial JA insensitivity (Navarro et al., 2008; Hou et al., 2010).

To further analyze the role of DELLAs on these hormone-related gene expression changes, we similarly tested the GSL gene expression of *ga1–3* in the response to JA and GA. Treatment with GA alone did not significantly affect the expression of JA-inducible genes (Figures 5A–F), but treatment with MeJA induced these genes to the same extent both in *ga1–3* mutant and in Col-0. However, in contrast with Col-0, combined GA/MeJA treatment did not strongly reduce the expression of JA-inducible genes but even led to moderately increased expression of *MYB34*. This indicates that the degradation of DELLAs in *ga1–3* probably also releases other bound interaction partners, such as MYC proteins besides the JAZ proteins (Figure 1D).

**Figure 6:**
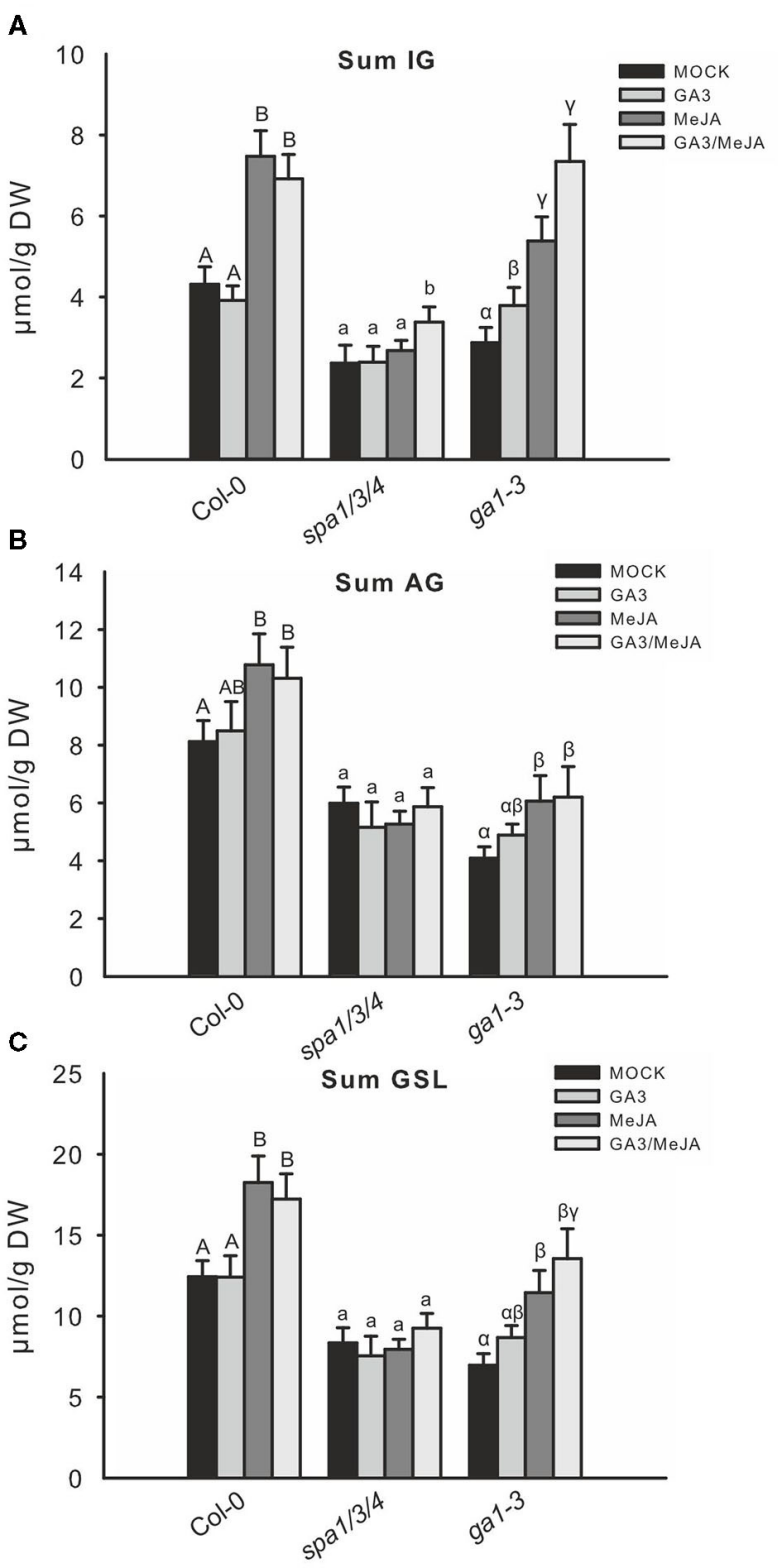
Combined GA/JA treatment further increases the IG level in *spa1/3/4* and *ga1–3* mutants. The effect of combined GA/JA treatment on GSL accumulation was tested in 6-week-*old spa1/3/4, ga1–3*, and Col-0 wild-type plants sprayed with MOCK, 50 μM GA_3_, 50 μM MeJA, or 50 μM MeJA/50 μM GA_3_ treatments. Data **(A–C)** are means ± SE from five independent experiments with four to six biological replicates in each case (*n* = 25). Different letters indicate significant differences (the Kruskal—Wallis test followed by a Mann–Whitney pairwise test with Bonferroni-corrected *p*-values, *p* < 0.05).

The accumulation of GSLs in wild type was consistent with changes in observed gene expression. Treatment with GA did not affect GSL accumulation but JA treatment led to a strong increase in the level of GSL, especially IG (Figure 6). Although combined GA/JA treatment led to decreased IG gene expression than with JA alone, the IG levels were hardly lower than those in response to JA alone. The *spa1/3/4* mutant did not respond by changing GSL accumulation after JA and GA treatments and only the IG content increased following GA/JA treatment. By contrast, the low GSL chemotype of *ga1–3*, whose phenotype results from high DELLA levels due to impaired GA biosynthesis, was partially complemented by GA treatment. Treatment with JA led to a greater IG induction and combined GA/JA treatment resulted in the highest IG level. The total IG level increased not significantly following GA/JA treatment; however, the level of I3M, which is JA-inducible, increased from 3.2 to 5.1 μmol/g DW. 4MO-I3M, which is usually repressed upon JA, was decreased from 1.4 to 1.0 μmol/g DW (Supplementary Figure 4). This increase was due to the GA-induced degradation of DELLA proteins since no increase was observed in the GA-insensitive DELLA-accumulating *gai mimic* mutant (Supplementary Figure 5).

**Figure 7:**
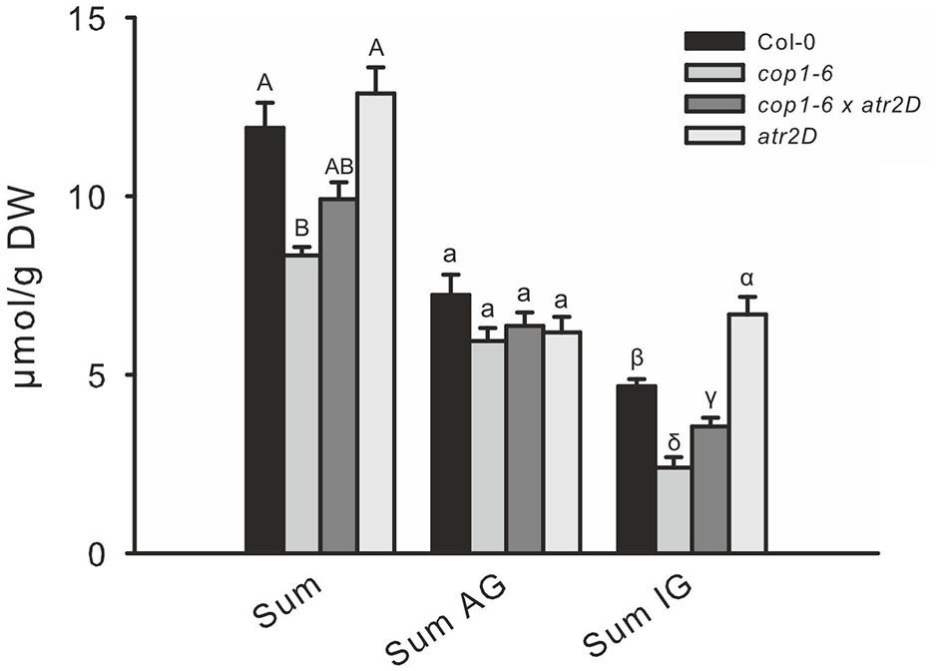
The release of MYC3/ATR2 from inactive complexes partially complements low indole GSL phenotype of *cop1*. To release MYC3 from inactive complexes in *cop1–6*, the *atr2D* gain-of-function line was combined with *cop1–6* to compare the GSL levels with that in *cop1–6*. Data are means ± SE from four independent experiments with five or six biological replicates in each (*n* = 22). Values marked with different letters differ significantly from each other (the Kruskal-Wallis test followed by a Mann–Whitney pairwise test with Bonferroni-corrected *p*-values, *p* < 0.05).

To test the hypothesis of whether a high level of DELLA proteins caused MYC inactivation and might thus be responsible for the low GSL phenotype in such DELLA-accumulating mutants, we crossed the constitutively active MYC3 allele (*atr2D*) with *cop1–6*. The release of MYC3 from inactive complexes partially complemented the low GSL phenotype (Figure 7).

## Discussion

### The Production of GSL in Arabidopsis Is Modulated by the Interaction Between COP1/SPA and DELLA Proteins

In this study, we investigated the role of COP1/SPA and DELLAs and identified them as new components in the regulation of GSL biosynthesis. Requirement of these upstream regulatory components seems to be inevitable upon dynamic environmental conditions as, among others, the production of GSL can be metabolically costly. Thus, flux balance analysis has estimated that the production of defense compounds requires a significant investment of energy and increases photosynthetic requirements by at least 15% in Arabidopsis (Bekaert et al., 2012). Thus, studies with Arabidopsis recombinant inbred lines (Paul-Victor et al., 2010) and with GSL knockout mutants at the early developmental stage (Züst et al., 2011) showed negative correlations between relative growth rates and higher GSL production. However, these effects disappeared during the plant life cycle, with a variable effect that depended on the type of GSL.

Molecular decisions made to coordinate growth and defense are determined by the abiotic environment including, in the case of GSLs, the availability of nutrients (Burow and Halkier, 2017), and also by light (Huseby et al., 2013). Light is an important signal for the allocation of plant resources to growth or defense. The absence of the main phyB photoreceptor in the *jazQ* mutant uncouples growth and defense responses by stimulating GA signaling and especially by activating PIF TFs (Campos et al., 2016). To investigate how GSL synthesis is affected by light and to uncover the molecular mechanism underlying phyB-dependent effects on the hormonal network for the growth–defense trade-off, we analyzed three different mutants in the COP1/SPA complex. Notably, all *cop* and *spa* mutants exhibited reduced growth (Supplementary Figure 2) and strongly reduced GSL synthesis (Figure 2A), which is consistent with reduced JA and GA signaling (Figures 2D,E). A yeast two-hybrid screen identified the DELLA protein GAI to be an interaction partner of SPA1 (Blanco-Touriñán et al., 2020), which could be confirmed by *in vivo* analysis. Arabidopsis DELLA proteins are key repressors of GA signaling and they serve as crucial convergence nodes of many signal transduction pathways that integrate hormonal responses and environmental stimuli (Claeys et al., 2014). The Arabidopsis genome encodes five DELLA proteins with distinct but redundant functions: GAI, RGA, RGL1, RGL2, and RGL3. In the absence of GA, DELLA proteins accumulate and repress GA responses, whereas, in the presence of GA, DELLA proteins are polyubiquitinated by the SCF^SLY1/GID2^ complex and are subsequently degraded by the 26S proteasome, thereby triggering GA signaling (Davière and Achard, 2013). Analogously, the interaction between the COP1/SPA complex and DELLA proteins can subsequently induce ubiquitination and degradation (Blanco-Touriñán et al., 2020). During night or shade conditions, COP1 enters the nucleus and actively represses TFs *via* ubiquitination (Hoecker, 2017). The absence of COP1 thus causes an increase in DELLA abundance during the night (Blanco-Touriñán et al., 2020).

### Does Inactivation of COP1/SPA Complex Repress MYC Proteins and Reduce GSL Synthesis *via* DELLAs?

The phenotype of other DELLA-accumulating mutants resembled that of *cop1* mutants in terms of GSL accumulation and the expression of JA/GA marker-genes (Figure 3). DELLA proteins interact with and inhibit JAZ protein function (Hou et al., 2010) and JAZ proteins, in turn, interact with and inhibit MYC function. Thus, competition among DELLA proteins to interact with JAZs might lead to an increase in active MYC proteins, but the opposite regulation is observed in *cop1* and other DELLA-enriched mutants (Figures 2, 3). However, DELLA proteins additionally interact with MYC2 (Hong et al., 2012; Yazaki et al., 2016) and were shown to inhibit MYC2 function in the regulation of sesquiterpene biosynthesis. Therefore, JA and GA are necessary for the full activation of sesquiterpene biosynthesis (Hong et al., 2012). Correspondingly, the low GA and JA phenotype in *cop1* and other DELLA-enriched mutants is presumably due to DELLA-inactivated bHLH TFs from the MYC and PIF subgroups.

Recent studies have shown that the three MYC proteins can be stabilized by red or blue light, whereas darkness and FR light promote their degradation (Chico et al., 2014). Furthermore, phytochromes and cryptochromes are required for MYC protein stability, and correspondingly, the destabilization of the MYC proteins in the dark is dependent on COP1. This suggests that despite lower *MYC2* expression (Figure 2E; Supplementary Figure 6), the *cop1–4* mutant contains a higher level of MYC2 protein (Chico et al., 2014). Nevertheless, the expression of MYC-dependent target genes is strongly reduced in *cop1* (Figures 2B–D; Supplementary Figure 6). This can be explained by the phenomenon known as “activation by destruction mechanism,” which was previously reported for MYC2 (Zhai et al., 2013) and implies that MYC2 can function as a transcriptional activator if it undergoes turnover while it is phosphorylated and subsequently degraded. Therefore, the high level of MYC2 protein and the low JA signaling phenotype in *cop1–4* can be explained by the absence of MYC protein activation and subsequent MYC degradation. Introduction of the dominant gain-of-function allele of MYC3 (*atr2D*) (Frerigmann et al., 2014; Goossens et al., 2015) into the *cop1–6* background largely rescued the low IG phenotype (Figure 7), pointing that the gain-of-function of MYC3, which can potentially enforce the accumulation of active MYC3 protein, is capable of rescuing the low GSL chemotype of *cop1* mutants.

Similarly, a reduction in the level of DELLA proteins in *cop1–4* in the absence of *RGA/GAI* rescued the low JA and GA phenotype partially (Figure 4; Supplementary Figure 3). Furthermore, the low degree of GSL and JA signaling in the DELLA-enriched mutant *ga1–3* was mostly rescued by the absence of four DELLA proteins (Figure 3), as shown by the expression of JA marker genes (*PDF1–2, LOX2*, and *TAT1*) in the *ga1–3/rga/gai/rgl1/rgl2* mutant (Hou et al., 2010).

### Light Perception and the Induction of Secondary Defense Compounds Are Linked to a Higher Level of DELLA Proteins and MYC Inactivation

In this study, we propose that light-activated photoreceptors inhibit the function of the COP1/SPA complex, which subsequently leads to higher levels of DELLA proteins; these DELLA proteins, in turn, inhibit and stabilize MYCs proteins by physical interaction (Figure 1C). This model is an add-on to the prevailing one as reported in previous studies (Navarro et al., 2008; Hou et al., 2010; Yang et al., 2012; Huot et al., 2014), in which JAZ-DELLA interactions cause reciprocal antagonism of GA and JA signaling. The updated model in Figure 1C makes clear that the effects of JAZ-DELLA interaction can be overridden, when COP1/SPA regulates the DELLAs, affecting in this way both the growth as well as biosynthesis of GSLs. This hypothesis is supported by observations that transferring plants from short days (SD) to long days (LD) increases resistance to *Botrytis cinerea* (Cagnola et al., 2018). The defensive secondary compounds required for resistance against this necrotrophic pathogen include camalexin and other indolic-derived secondary compounds, which are synthesized similarly to the IGs addressed in this study. The perception of a longer light period, which activates the synthesis of these compounds and results in improved plant resistance, is dependent on the photoreceptors phyA, cry1, and cry2, whereas exposure to short days decreases MYC2 stability in a COP1-dependent manner. Correspondingly, a longer light period inhibits the COP1/SPA complex and increases nuclear DELLA abundance (Cagnola et al., 2018). Thus, DELLA proteins interact with, inhibit, and stabilize MYC2. Accordingly *myc2* and *cop1* mutants show enhanced resistance to *B. cinerea* in SD (Cagnola et al., 2018). Contrary to the increased *B. cinerea* resistance following inactivation of COP1 and MYC2 by LD (compared with SD), shade conditions (FR light treatment) increased susceptibility to *B. cinerea* (Cerrudo et al., 2012) by triggering the inactivation of phyB (a COP1/SPA repressor). Therefore, the *phyB* mutant was highly susceptible to *B. cinerea* without FR treatment (Cerrudo et al., 2012). FR light and *phyB* mutation trigger DELLA degradation and increase JAZ10 stability (Leone et al., 2014), which is consistent with a higher activity of the COP1/SPA complex.

Exposure to FR light increases susceptibility to *B. cinerea* due to reduced JA sensitivity, resulting in lower IG and camalexin levels, and this is additionally controlled by JAZ10, because the *jaz10* loss-of-function mutant lacks FR light-dependent reduction in IG in normal conditions and following MeJA treatment (Cargnel et al., 2014). Furthermore, the stability of most JAZ proteins is enhanced by FR light, which delays JA-dependent JAZ degradation (Chico et al., 2014). However, the absence of JAZ10 function appears to repress many of the defense phenotypes in the *phyB* mutant, such as low IG content, high susceptibility to *B. cinerea*, and low JA-inducible gene expression, but not the shade avoidance phenotypes (Cerrudo et al., 2017). This indicates that a low level of DELLA proteins together with decreased abundance of JAZ proteins might not strongly repress GA signaling, including shade-avoidance responses by PIF proteins.

### The Ratio of DELLA–MYC, DELLA–JAZ, and JAZ–MYC Inactive Complexes Might Depend on the Relative Different Stoichiometric Abundances of Their Components in Diverse Tissues and Following Biotic/Abiotic Stimuli

The degradation of DELLA proteins in the presence of GA releases JAZ proteins to attenuate MYC2 function (Hou et al., 2010). Combined treatment with MeJA and GA strongly reduced the expression of JA-induced genes compared to treatment with JA alone (Figure 5; Supplementary Figure 7). However, treatment with GA alone did not repress these genes. The production of GSLs, especially IG, was strongly induced by JA but was hardly affected by combined GA/JA treatment or GA alone. However, treatment of the DELLA-accumulating mutant, *ga1–3*, with GA increased the level of JA-inducible IG (Figure 6; Supplementary Figure 7), indicating that the resulting decrease in DELLA protein abundance releases MYC proteins from inactive complexes. Similarly, combined JA/GA treatment did not lead to a reduction in GSL content compared with JA treatment alone, as in Col-0, but the content of the JA-inducible IG, I3M, increased strongly (Supplementary Figure 4). Compared to the reduced expression of JA-inducible genes in Col-0, combined GA/JA treatment led to a slight increase or at least stable transcript levels of most JA-inducible GSL regulatory or synthesis genes in *ga1–3* (Figure 5). Thus, in a DELLA-enriched mutant, the GA-induced degradation of DELLA proteins in addition to the JA-induced degradation of JAZ proteins might release even more MYC proteins from inactive complexes than JA treatment alone. Similarly, the *spa1/3/4*, which accumulates DELLA proteins, showed a higher content of IG following combined JA/GA treatment compared with treatment with JA alone (Figure 6; Supplementary Figure 7). The greater response of *ga1–3* than *spa1/3/4* to combined GA/JA treatment indicates that the COP1/SPA complex might affect GA and JA signaling in a much more complex manner. For example, MYC2-mediated abscisic acid (ABA) and JA responses are further modulated by SPA1 (Gangappa et al., 2010), and JA stabilizes several COP1-targeted TFs in a COP1-dependent manner (Zheng et al., 2017). Another study has identified a sulfotransferase (ST2a) recently and showed that it is strongly upregulated by plant proximity perceived by phyB (Fernández-Milmanda et al., 2020). By catalyzing the formation of a sulfated JA derivative, ST2a acts to degrade bioactive forms of JA and represents another molecular link between photoreceptors and hormone signaling in plants.

## Conclusions

We have uncovered another crucial component of the hardwired hormone-linked transcriptional network upstream from the MYC–bHLH TF complex, which regulates the production of defensive GSL compounds (Figure 1; Supplementary Figure 7). The COP1/SPA complex is a central nexus that integrates light perception *via* phyB and other photoreceptors and transforms several interconnected components into a finely balanced system of growth and defense. The COP1/SPA complex positively regulates growth at an additional level *via* interacting with and ubiquitinating DELLA proteins in shade or night conditions (Blanco-Touriñán et al., 2020).

The COP1/SPA complex also interacts with PIF proteins (Zhu et al., 2015) and is critical to maintaining high GA levels in varying light conditions (Weller et al., 2009). The DELLA proteins themselves constitute a crucial convergence point of many signal transduction pathways that are triggered by hormones and environmental stimuli (Claeys et al., 2014). We show that different DELLA-accumulating mutants not only inhibit JAZ repressors but strongly repress general JA signaling *via* MYC interaction (Figure 1; Supplementary Figure 7). GSL synthesis can only be fully activated by simultaneous activation of GA and JA pathways, a situation that resembles the induction of sesquiterpene biosynthesis in inflorescences (Hong et al., 2012). Inflorescences also possess a high level of DELLA protein expression (Lee et al., 2002; Tyler et al., 2004). Thus, the observed inactivation of MYC proteins by DELLA proteins appears to represent an additional layer of regulation that depends on the stoichiometric ratio of DELLA and JAZ proteins in specific tissues or cells. Such a multi-tiered mechanism to powerfully repress the expression of chemical defenses might prevent deleterious effects of carbon depletion or metabolic imbalance during the growth-to-defense transition (Guo et al., 2018). In specific tissues and situations that led to a high abundance of DELLA proteins, this additional layer of regulation might safeguard against too many resources being allocated to defense in the absence of a specific trigger. Overall, independent of molecular mechanisms behind growth–defense trade-off under given environmental conditions, the whole ontogeny of the organisms and their environmental context needs to be taken into account. The production of defense compounds does not necessarily incur net ecological costs, because the metabolic requirements for defense are not automatically the same as for those for growth (Kliebenstein, 2016; Mitreiter and Gigolashvili, 2021).

In summary, these findings highlight the importance of COP1/SPA and DELLA upstream regulatory components in balancing the defense and growth. However, growth restriction does not seem to be a direct consequence of defense activation but rather a part of a defense system, which is adaptive under conditions of a biotic attack and maladaptive under conditions where defense is not the major functional priority for survival (BallaréBallaré and Austin, 2019). The complexity of this pathway is also highlighted in contrasting observations made during the analysis of *phy jaz* mutants. While *phyB jazQ* plants capable of uncoupling growth and defense as PIF and MYC TFs are getting simultaneously activated (Campos et al., 2016), the *phyB jazD* is unable to completely uncouple growth-defense phenotypes (Major et al., 2020). Notably, the abundant RGA protein was not increased in untreated *jazQ* or *jazD* seedlings relative to WT (Major et al., 2020), suggesting that either (i) change in DELLA protein activity does not play a major role in restricting the shoot growth of *jaz* mutants or (ii) that the DELLA pathway (e.g., yet to be explored regulatory RNAs or small signaling metabolites) is involved in this process but not the DELLA proteins.

Thus, the molecular mechanism of JA-induced biomass reduction and the potential role of DELLAs and MYCs in this process continues to be thrilling. Not only the role of the DELLA pathway beyond its activity on protein level but also the role of MYCs in the regulation of plant growth-defense balance needs to be addressed in the future in more detail. Exploration of other signaling options, including small regulatory RNA and metabolite signals, is a must to find the solution for growth-defense conflicts in plants. While progress has been made to identify some hard-wired genetic and ecophysiological regulators that constrain plant growth and development during immune responses, recent advances in disciplines like systems biology, bioinformatics, and machine learning will open the gate for the identification of missing regulatory mechanisms balancing the plant growth and immunity.

## Data Availability Statement

The original contributions presented in the study are included in the article/Supplementary Material, further inquiries can be directed to the corresponding author/s.

## Author Contributions

HF designed the experiments, performed most of the experiments, analyzed the data, and wrote the manuscript. TG performed qPCR experiments and was actively involved in the writing and publication process. UH contributed to the experimental design and the writing. All authors contributed to the article and approved the submitted version.

## Conflict of Interest

The authors declare that the research was conducted in the absence of any commercial or financial relationships that could be construed as a potential conflict of interest.
