# Supplementary material for: New Insights on the Regulation of Glucosinolate Biosynthesis via COP1 and DELLA Proteins in Arabidopsis Thaliana
Source: Front Plant Sci. 2021 Jul 1;12:680255. doi: 10.3389/fpls.2021.680255 (PMC8281118; doi:10.3389/fpls.2021.680255)
Supplement: Supplementary file 1 [file Data_Sheet_1.PDF]

-Supporting information

Supplemental Figures

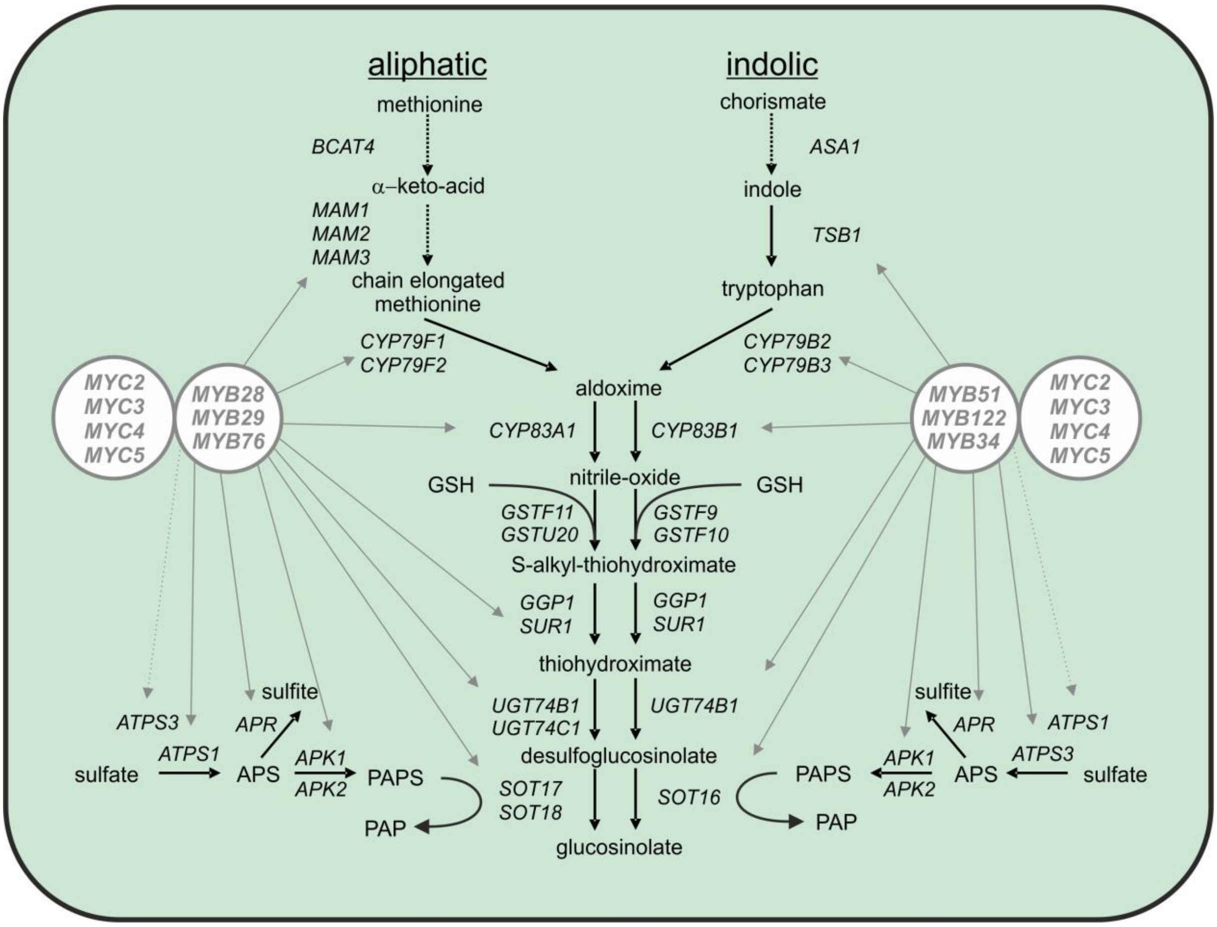

Suppl. Fig. 1. Schematic overview of the transcriptional regulation of aliphatic and indole glucosinolate biosynthesis genes by MYB and MYC transcription factors

The biosynthesis of AG and IG share many enzymes in the core pathway, with partially overlapping affinities to their substrates. All depicted GSL biosynthesis genes are regulated by MYB–MYC complexes (reviewed by Frerigmann (2016)). The displayed arrows present the demonstrated *trans*-activation ability of the respective HIG- or HAG-MYB transcription factors (Yatusevich et al. 2010). Figure is taken from Frerigmann (2016).

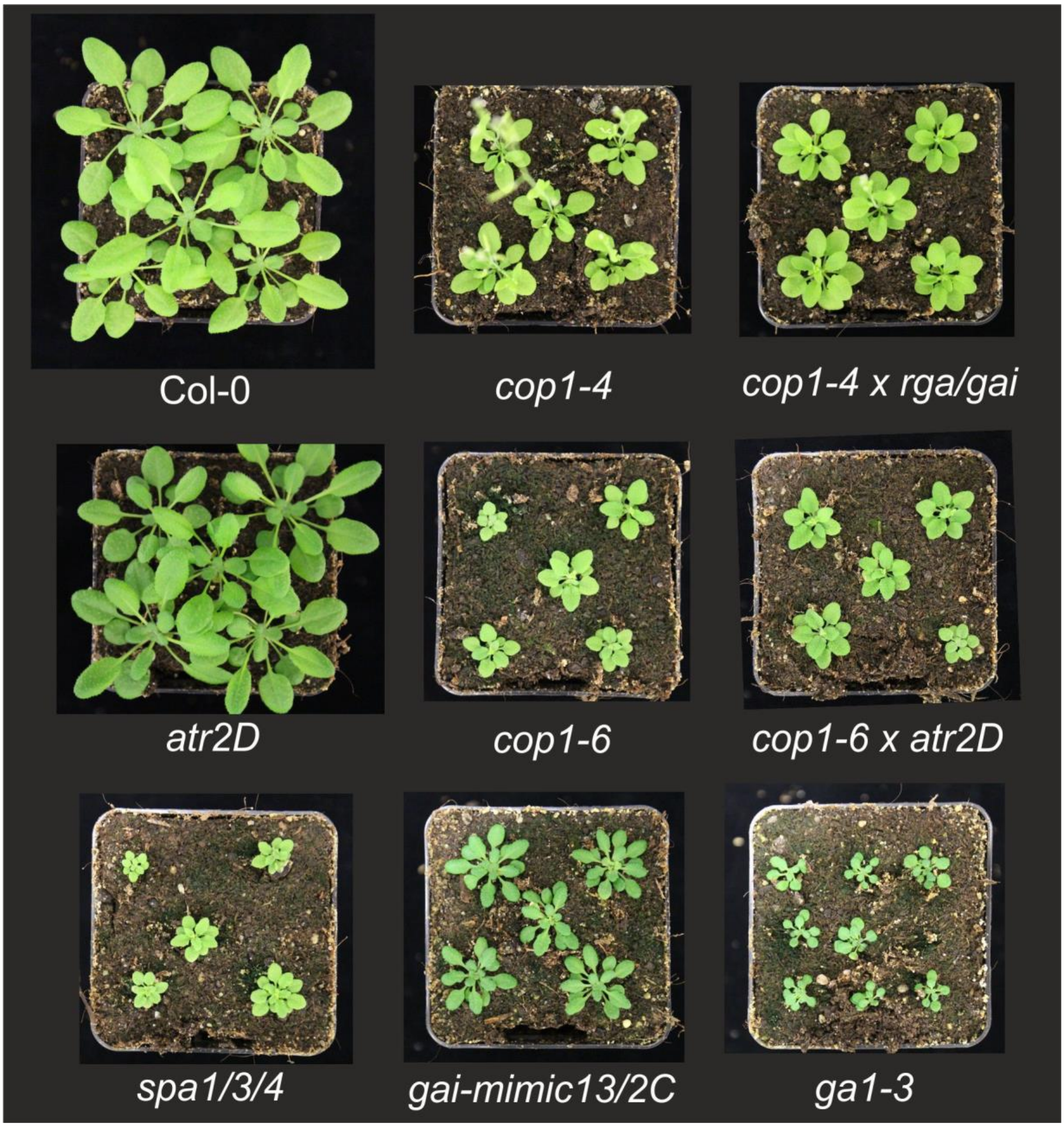

Suppl. Fig. 2. Growth phenotype of 6-week-old *cop1-4, cop1-4 x rga/gai, atr2D, cop1-6, cop1-6 x atr2D, spa1/3/4, gai-mimic13/2c* and *gai1-3* mutant plants grown under SD conditions

All *cop* and *spa* mutants exhibited reduced growth under this SD conditions, but the absence of only two DELLA proteins was sufficient to partially complement the *cop1-4* phenotype and led to larger leaves, more rosette leaves and later flowering

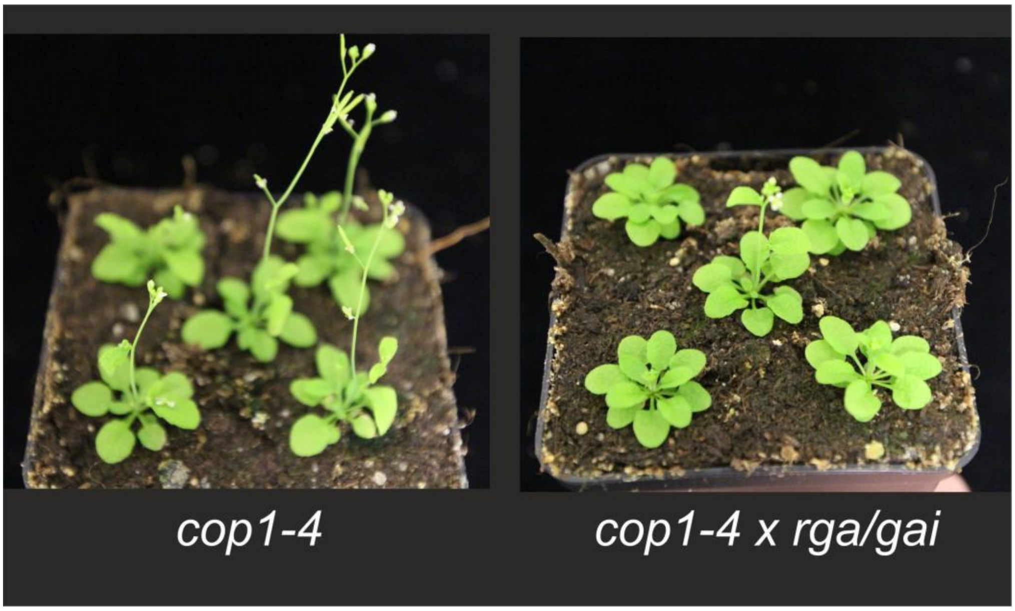

Suppl. Fig. 3. The *cop1-4/rga1/gai* mutant flowers later and has more and larger rosette leaves than *cop1-4*

Growth phenotype of 6-week-old *cop1-4* and *cop1-4 x rga/gai* mutant plants grown under SD conditions.

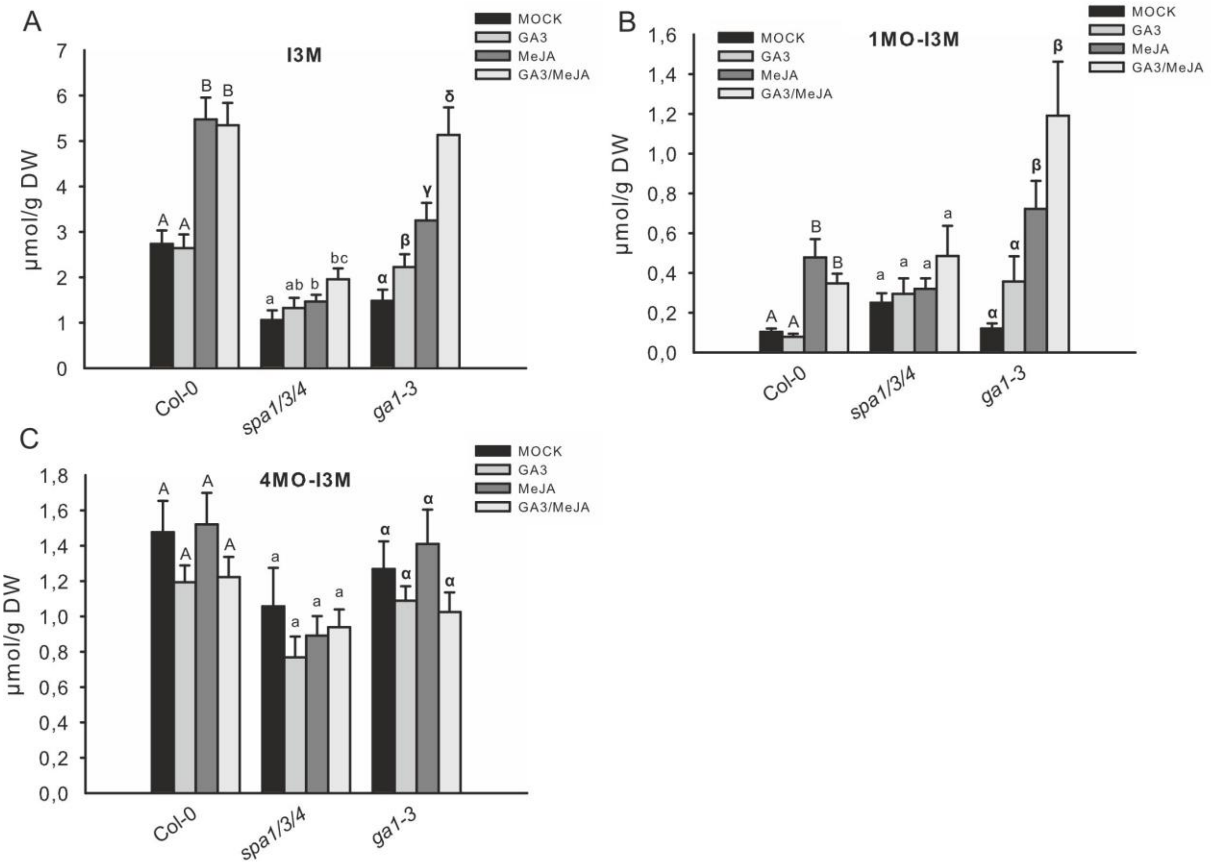

Suppl. Fig. 4. Combined GA/JA treatment further increases the IG level in *sp1/3/4* and *gai1-3* mutants

The effect of combined GA/JA treatment on GSL accumulation was tested in 6-week-old Col-0 wild-type plants sprayed with a MOCK, 50 µM GA_3, 50 µM MeJA or 50 µM MeJA/50 µM GA_3 treatment. Data are means ± SE from five independent experiments with four to six biological replicates in each (n

= 25). Different letters indicate significant differences compared to each MOCK (Kruskal-Wallis test followed by a Mann-Whitney pairwise test with Bonferroni-corrected p-values $p < 0.05$).

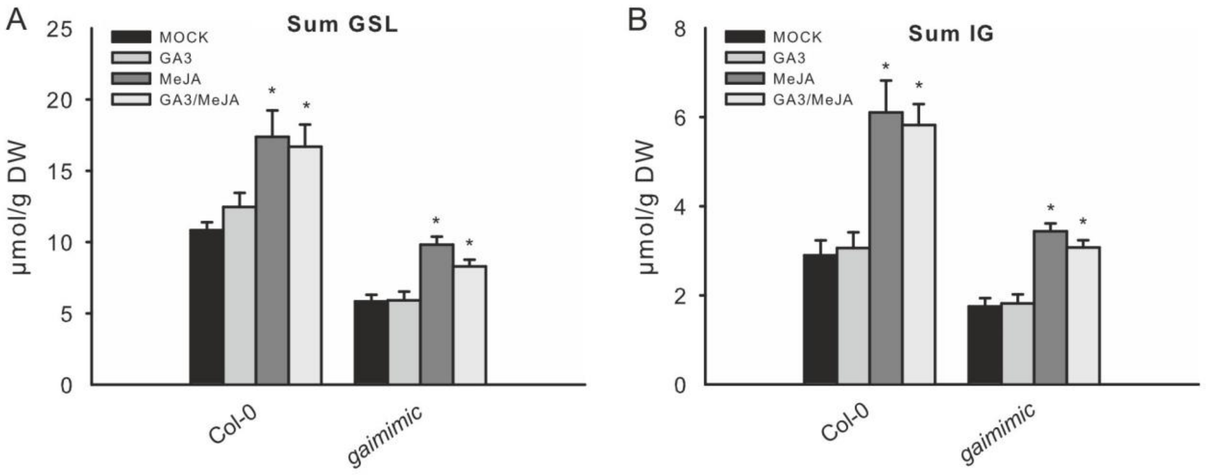

Suppl. Fig. 5. Combined GA/JA treatment did not further induce GSL levels in the GA-insensitive *gai mimic* mutant

The effect of combined GA/JA treatment on GSL accumulation was tested in 6-week-old plants sprayed with a MOCK, 50 µM GA_3, 50 µM MeJA or 50 µM MeJA/50 µM GA_3 treatment. Data are means ± SE from two independent experiments with six biological replicates in each (n = 12). Values marked with asterisks differed significantly from Col-0 (Student's t-test; $p < 0.05$).

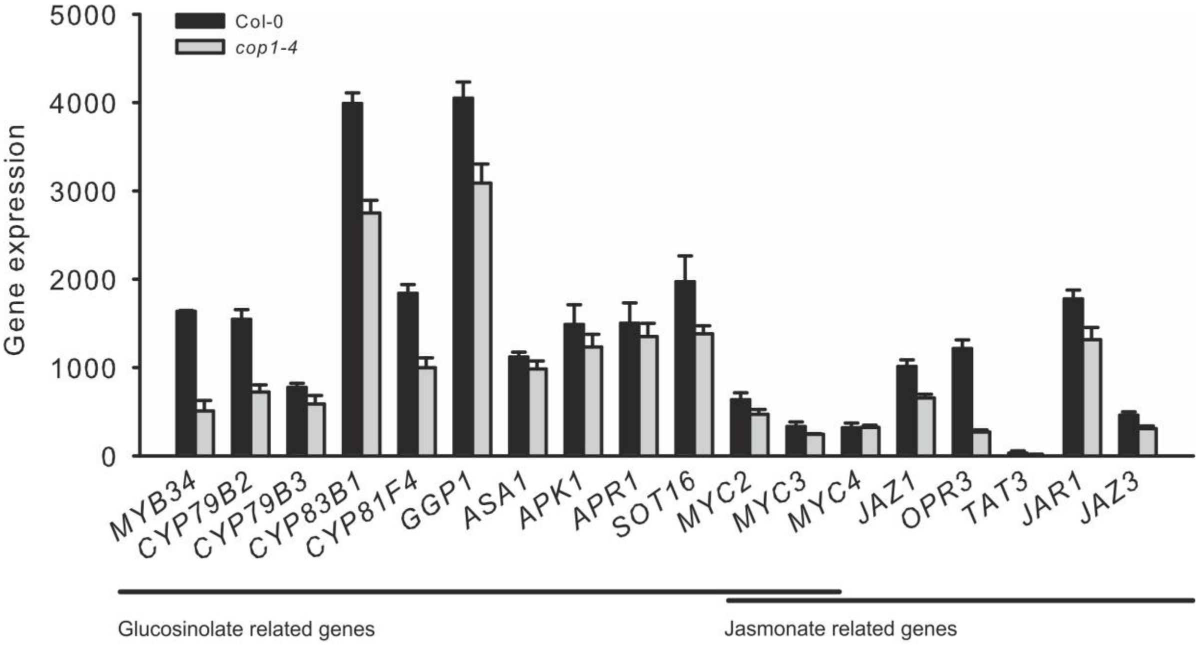

Suppl. Fig. 6. The *cop1-4* mutant shows reduced expression of many glucosinolate- and jasmonate-related genes

Selected genes from publicly available microarray experiments (Pacín et al. 2016) in Col-0 and *cop1-4* are shown. Samples were harvested 5 h after the beginning of Day 3.

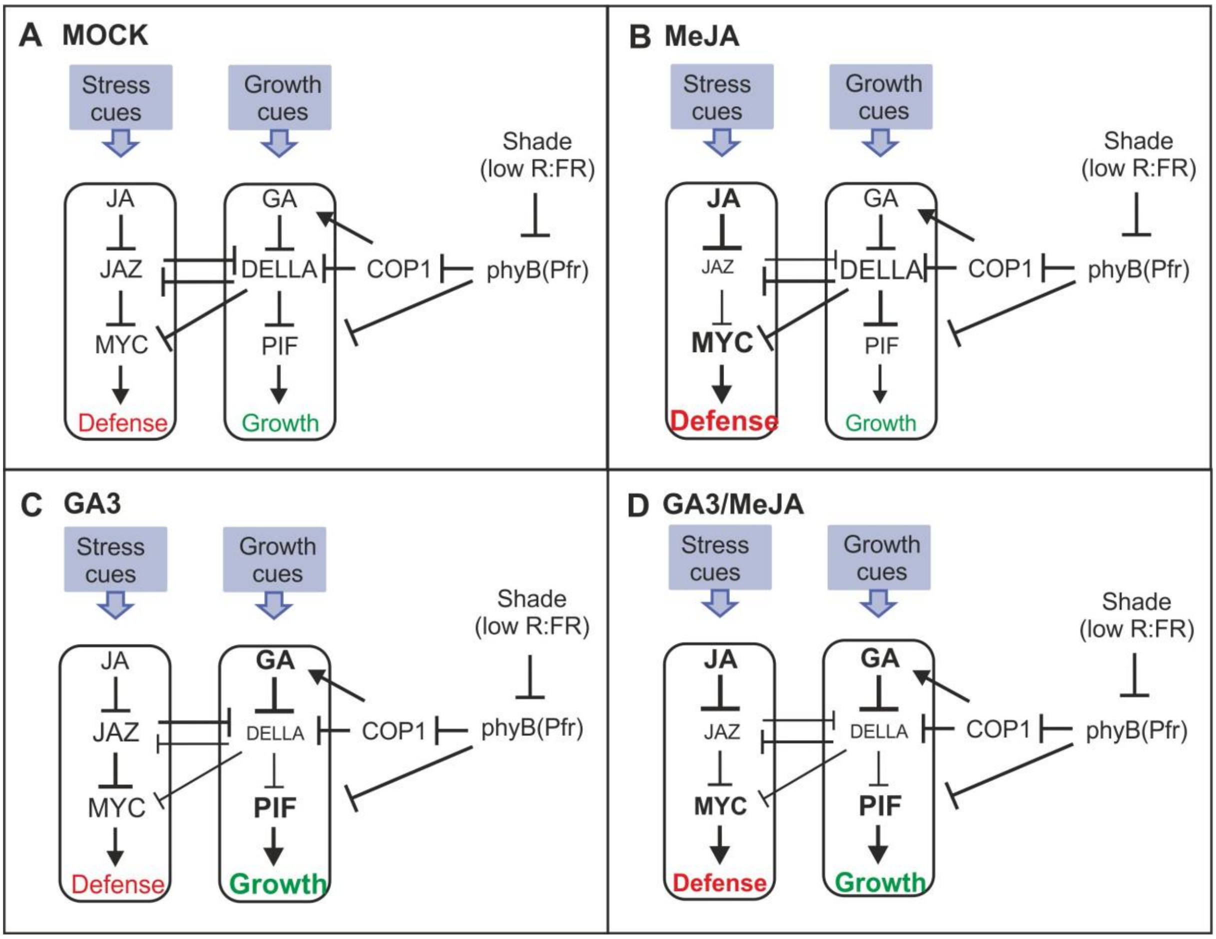

Suppl. Fig. 7. Simplified model for the effects of JA and GA treatment on the JAZ–DELLA network that links growth and immunity tradeoff

A revised model of the JA/GA signaling network that governs growth and defense (altered from Campos et al. (2016). The figure depicts the effect of MeJA and GA_3 treatment on Col-0 wild-type plants described in **Fig. 5** and **Fig. 6.** Differently sized letters and arrows indicate proposed abundances and activities. MOCK (**A**). Treatment with 50 µM MeJA represses JAZ abundance and thereby releases MYC proteins from inactive complexes, which in turn, activate defense responses *inter alia* IG production. Furthermore, due to lower JAZ levels, more DELLA proteins are available to repress the growth response e.g., via PIF proteins (**B**). Spray-treatment with 50 µM GA_3 leads to the degradation of DELLA proteins and the release of PIF proteins, which in turn, induce growth. Furthermore, DELLA protein degradation provides more free JAZ proteins, which can repress MYC proteins; however, the repressing effect of DELLA proteins on MYC proteins might also be reduced, leading to a slight increase in some JA responses (**C**). Combined treatment with GA_3 and MeJA induces the degradation of JAZ and DELLA proteins and in turn, activates MYC and PIF proteins to induce defense and growth. However, in comparison to MeJA treatment alone, MYC2 activation is repressed, which might be due to the release of JAZ proteins from JAZ–DELLA complexes (**D**).

Supplemental Tables

Suppl. Table 1. Primer sequences for qPCR analysis

Oligonucleotide name	AT-Number	Oligonucleotide sequence 5´–3´
PP2A_RL_Fw	AT1G59830	CAAGAGGTTCCACACGAAGGA
PP2A_RL_RV		TGTAACCAGCACCACGAGGA
CYP83A1_RL_Fw	AT4G13770	TTCAAGAGGTTGTCAATGAGACGC

CYP83A1_RL_Rv		CTACAATATCCAAGATGACGGCTTT
CYP79B3_RL_Fw	AT2G22330	CTCCTTCTTCCTTGCAAATGGA
CYP79B3_RL_Rv		GAGAATCATCAAGAAGCAAAGGG
CYP83B1_RL_Fw	AT4G31500	GGCAACAAACCATGTCGTATCAAG
CYP83B1_RL_Rv		CGTTGACACTCTTCTTCTCTAACCG
MYB34_RL_Fw	AT5G60890	CACGACTGTCGATAATTTTGGGTT
MYB34_RL_Rv		CATATTGTCATCTTCGTTCCAGGA
MYB51_RL_Fw	AT1G18570	CTACAAGTGTTTCCGTTGACTCTGAA
MYB51_RL_Rv		ACGAAATTATCGCAGTACATTAGAGGA
MYB122_RL_RV	AT1G74080	AACTTCATTGATCGGCGTCAC
MYB122_RL_Fw		ACCTCTTCGAATCTCCCCATC
MYC4_RL_Fw	AT4G17880	AGAAGAGTTGCAGAAGCAGATTGA
MYC4_RL_Rv		GATCTTTTACCGAACTTTTCGCAT
MYC3_RL_Fw	AT5G46760	TTGGGATGTGATGATACGTGTACA
MYC3_RL_Rv		TTAAGTGCTTCCATGAACCTAGCA
MYC2_RL_Fw	AT1G32640	ATGTCGTCTTCGTGTTCTTCGAT
MYC2_RL_Rv		GCGTCCCAACCAATTATCTTCACT
JAZ1_RL_Fw	AT1G19180	AGCTTCACTTCACCGGTTCTTGGA
JAZ1_RL_Rv		TCTTGTCTTGAAGCAACGTCGTCA
PRE5_RL_Fw	AT3G28857	AGATGATCGACCTCGTTAGTAAGC
PRE5_RL_Rv		TACCTTTGATGCTGACACCTTATC
PR1_RL_Fw	AT2G14610	GTGCCAAAGTGAGGTGTAACAA
PR1_RL_Rv		CGTGTGTATGCATGATCACATC
VSP2_RL_Fw	AT5G24770	CTCTTGGTCGCTACGGTCTC
VSP2_RL_Rv		GGCACCGTGTCGAAGTTTAT

Campos, M. L., Y. Yoshida, I. T. Major, D. de Oliveira Ferreira, S. M. Weraduwage, J. E. Froehlich, B. F. Johnson, D. M. Kramer, G. Jander and T. D. Sharkey (2016). "Rewiring of jasmonate and phytochrome B signalling uncouples plant growth-defense tradeoffs." Nature communications 7: 12570.

Frerigmann, H. (2016). "Glucosinolate Regulation in a Complex Relationship - MYC and MYB - No One Can Act Without Each Other." Advances in Botanical Research 80: 57-97.

Pacín, M., M. Semmoloni, M. Legris, S. A. Finlayson and J. J. Casal (2016). "Convergence of CONSTITUTIVE PHOTOMORPHOGENESIS 1 and PHYTOCHROME INTERACTING FACTOR signalling during shade avoidance." New Phytologist 211(3): 967-979.

Yatusevich, R., S. G. Mugford, C. Matthewman, T. Gigolashvili, H. Frerigmann, S. Delaney, A. Koprivova, U. I. Flügge and S. Kopriva (2010). "Genes of primary sulfate assimilation are part of the glucosinolate biosynthetic network in *Arabidopsis thaliana*." Plant J. 62: 1–11.
